# Suture-related pseudoinfection after total hip arthroplasty

**DOI:** 10.1007/s10195-014-0300-4

**Published:** 2014-06-11

**Authors:** Luca Pierannunzii, Andrea Fossali, Orazio De Lucia, Arturo Guarino

**Affiliations:** Gaetano Pini Orthopaedic Institute, P.zza Cardinal Ferrari, 1, 20122 Milan, Italy

## Abstract

Absorbable sutures are widely used for wound closure after total hip replacement. Here we present two cases of suture-related foreign-body reaction that perfectly mimicked a periprosthetic joint infection, with sterile abscess formation and physical and laboratory signs of inflammation acutely presenting 7–8 weeks after surgery, at the time of suture absorption. Both recurred with analogous timing after irrigation and debridement, likely due to re-using the same suture material. Multiple negative microbiological samples and positive histological samples showing a foreign-body reaction are the fundamental steps towards the diagnosis of a suture-related pseudoinfection (SRPI). Only three other cases have been reported to date, but the recurrence, together with the self-healing course after relapse, represents a completely novel feature and possibly the strongest demonstration of the supposed aetiopathogenesis. The knowledge of this possible complication leads to some clinical implications: all potential periprosthetic joint infections should routinely undergo not only microbiological but also histological sampling; caution should be used when recommending prosthesis exchange for potential infections occurring in the time range of suture absorption; lastly, if SRPI is suspected, a suture with low propensity to induce foreign-body reactions should be chosen after irrigation and debridement and the volume of absorbable material left in the wound should be as small as possible.

## Introduction

Infection is probably the most dangerous and feared complication after total hip arthroplasty (THA). Since timely treatment is mandatory to increase the chance of success, careful patient monitoring and prompt irrigation and debridement of possibly infected wounds are essential [1].

Absorbable sutures are widely used for wound closure after THA, and Vicryl Plus^®^ (Ethicon, Johnson & Johnson) combines the features of a well known absorbable suture (Vicryl^®^) with a broad-spectrum antibacterial agent (Triclosan).

A few cases of adverse reactions to Vicryl^®^/Vicryl Plus^®^ have reported to date [2] in contrast with the worldwide circulation of these products in most fields of surgery; however, interestingly, three cases were described as mimicking infection after THA [3].

The present paper aims to present another two cases, whose clinical history, histopathological and laboratory findings are so distinctive (and consistent with previous reports) as to define a novel, exceptional THA complication, the suture-related pseudoinfection (SRPI).

## Case report

### Case #1

A 63-year-old woman with displaced femoral neck fracture of the left hip underwent cementless ceramic-on-ceramic THA through straight lateral approach. The patient had no relevant risk factors for infection (immunocompetent, non-diabetic with normal body mass index and no history of recent infections) except light smoking (less than 10 cigarettes per day), and surgery was completed within 80 min. Antibiotic prophylaxis was obtained with a short intravenous course of cefazolin (2 g before operation, followed by 1 g 6–14–22 h later). The trochanteric digastrics tendon split and the fascial incision were sutured with Vycril Plus^®^ #2, while subcutaneous tissue was sutured with Vycril Plus^®^ #2 and #0 in the deep layer and Vycril Plus^®^ #2/0 in the superficial layer. Staples were used for skin closure. Two deep suction drains were maintained for 48 h and removed at first dressing change. The post-operative course was uneventful: body temperature normalized (below 37 °C) 2 days after surgery, the wound was dry with no signs of inflammation or hematoma, C-reactive protein (CRP) levels halved every 2 days, and the hip was mobile and pain-free. The patient was therefore discharged home 8 days after surgery. On the 14th postoperative day skin staples were removed and on the fifth week the patient was seen in the outpatient clinic; X-rays and clinical examination were extremely satisfactory, and she was allowed to abandon her crutches and to resume ordinary life activities.

In the ninth week from index surgery the patient, previously pain-free, started to complain of tenderness, warmth and redness of the skin around the scar. She was examined immediately after symptom onset and a minimal seropurulent discharge was noticed from a small sinus, which was carefully dilated with a sterile swab, allowing the exudate to drain and microbiological samples to be collected (with negative results). Blood tests detected mildly elevated CRP (1.4 mg/dL) and erythrocyte sedimentation rate (ESR) (60 mm/h), but no elevation of white blood cell (WBC) count. Ultrasonographic (US) examination of the hip demonstrated an abscess in the deep layer of the hypodermis, with several sinus tracts towards the surface. The presence of local signs (warmth, redness, swelling, tenderness and fluid discharge), US signs (abscess) and laboratory signs (elevated CRP) of surgical site infection convinced us to schedule immediate irrigation and debridement (ID) within 1 week from complication onset.

The debridement was performed through the pre-existing scar, with excision of multiple sinus tracts. A massive abscess, with purulent grey-yellowish content, was retrieved in the deep subcutaneous tissue, extending along the whole incision. After culture and histological sampling, the cavity was debrided and irrigated with diluted iodopovidone and saline solution. The fascia, apparently intact, was then incised and the pertrochanteric space was inspected. Since no signs of infection were retrieved below the fascia, surgical gowns, gloves and instruments were replaced before splitting the digastrics tendon and opening the periprosthetic capsule; within the joint just a few milliliters of clear fluid were found. Thorough irrigation was performed after microbiological sampling. Since the infection seemed not to have spread below the fascia, and given the risk of ceramic rupture associated with head and liner exchange, no attempt was made to remove them. The wound was closed in a standard fashion, but employing as few sutures as possible so as not to leave an excessive amount of foreign material in a potentially infected surgical site, and two suction drains (whose tips were sent to the microbiology laboratory for further cultures) were placed. During the procedure, immediately after culture sampling, an empirical course of antibiotics was started (teicoplanin 800 mg and levofloxacin 1 g i.v.) and was confirmed postoperatively (teicoplanin 600 mg q.d. and levofloxacin 500 mg b.i.d.).

On the first postoperative day the patient was already pain-free, her body temperature normalized and the wound healed regularly. CRP stayed within the range throughout the hospitalization, after normalizing with sinus drainage 3 days before surgery. No cultures were positive, but given the strong suspicion of infection and the absence of adverse reactions to antibiotics, the patient was discharged home 7 days after ID with an oral 4-week therapy (cotrimoxazole 800 mg/160 mg b.i.d. and levofloxacin 500 mg q.d.).

**Fig. 1:**
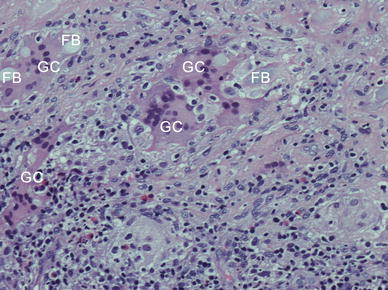
Foreign-body reaction in the superficial hypodermis. *GC* giant cell, *FB* foreign body (haematoxylin and eosin, original magnification 200×)

The histological examination of the collected material demonstrated a giant-cell foreign-body reaction, where some amorphous birefringent material was clearly visible (Fig. 1).

**Fig. 2:**
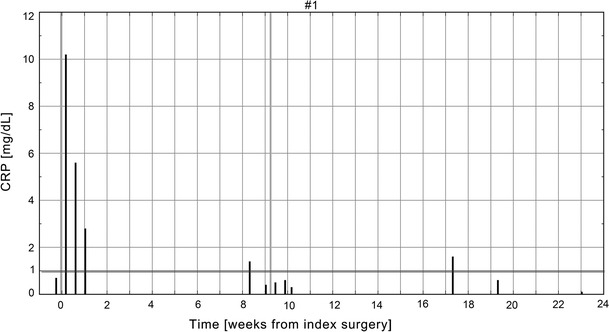
C-reactive protein kinetics of patient #1. Weeks are calculated from the index surgery (THA). The *two grey vertical lines* represent the procedures (THA and ID), while the *grey horizontal line* represents the highest value of the normal CRP range (1 mg/dL)

Even though the patient was asymptomatic, she was followed up monthly with physical examinations and blood tests (Fig. 2), and 8 weeks after ID another mild CRP elevation (1.6 mg/dL) was noticed without reasonable causes, except minimal scar inflammation and extrusion of suture material. The wound was treated with iodopovidone solution and daily dressing change and healed in a week after complete extrusion of the foreign material. CRP normalized and no further complications have occurred for over 20 months.

### Case #2

A 64-year-old woman affected by bilateral hip osteoarthritis underwent cementless ceramic-on-ceramic right hip THA through straight lateral approach. She had no relevant risk factors for infection, surgery was uncomplicated and lasted about 70 min. The same antibiotic prophylaxis, surgical technique and suture materials were employed as in case #1. The post-operative course was similarly uneventful: body temperature never exceeded 37 °C, the wound was dry with no signs of inflammation or hematoma, CRP fell within the normal range in 12 days, and the hip was mobile and pain-free. The patient was therefore discharged to the rehabilitation facility as soon as an inpatient rehab bed was available, 8 days after surgery. On the 15th postoperative day skin staples were removed, she returned home and on the fifth week the patient was seen in the outpatient clinic with excellent functional recovery and X-rays. She was allowed to abandon her crutches and to resume ordinary life activities.

In the eighth week after THA, almost as in case #1, the patient, previously pain-free, started complaining of tenderness, warmth and redness of the scar, with mild elevation of body temperature (37.5 °C). Ambulation became painful as well as lying on the operated side. She was seen 3 days after symptom onset and no drainage was noticed from the scar, but it was extremely painful on palpation. Blood tests detected mildly elevated CRP (1.5 mg/dL) and ESR (50 mm/h), but no WBC count elevation. Ultrasonographic examination of the hip demonstrated a bulky pertrochanteric abscess, with several sinus tracts perforating the fascia towards the surface. No joint effusion was clearly documented. The presence of local signs (warmth, redness, swelling and tenderness), US signs (abscess) and laboratory signs (elevated CRP) of surgical site infection persuaded us to schedule prompt reoperation for ID.

The debridement was performed through the pre-existing scar and a massive abscess, with purulent grey-yellowish material, was retrieved in the deep hypodermis. Several fistulae perforated the fascia and allowed the exudates to spread in the pertrochanteric space. After culture and histological sampling, the cavity was debrided and irrigated with diluted iodopovidone and saline solution. The fascia was then incised, trans-fascial fistulae excised and the pertrochanteric space debrided and irrigated similarly. Since the abductor mechanism seemed to be intact and the preoperative US examination did not show joint space effusion, surgical gowns, gloves and instruments were replaced before splitting the digastrics tendon and opening the joint capsule; the same healthy periprosthetic environment was found as in case #1. The procedure was completed as previously described, with microbiological sampling, careful joint irrigation but without head/liner exchange, and administering the same intravenous empirical antibiotic therapy.

On the first postoperative day the patient was already pain-free, with body temperature normalized. CRP normalized on the second day and the wound healed regularly. No cultures (either intraoperative or postoperative on drainage tube tips) were positive, but given the strong suspicion of infection and the absence of adverse reactions to antibiotics, the patient was discharged home 13 days after ID with an oral 4-week therapy (amoxicillin 1 g t.i.d. and levofloxacin 500 mg q.d.).

**Fig. 3:**
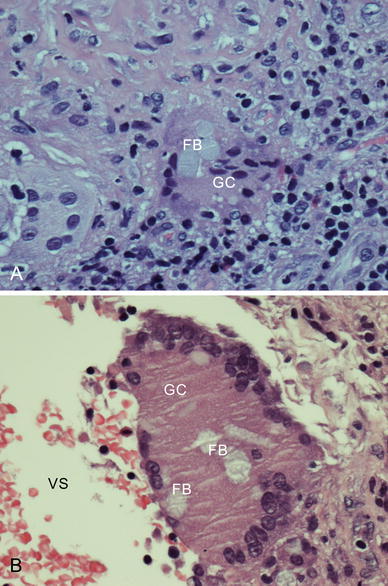
Foreign-body reaction in the superficial (**a**) and deep (**b**) hypodermis. *GC* giant cell, *FB* foreign body, *VS* vascular space (haematoxylin and eosin, original magnification 400×)

Histological examination of the material collected showed the same pattern of foreign-body reaction: a mixed inflammatory cell infiltrate, with multinucleated giant cells and amorphous birefringent material (Fig. 3).

**Fig. 4:**
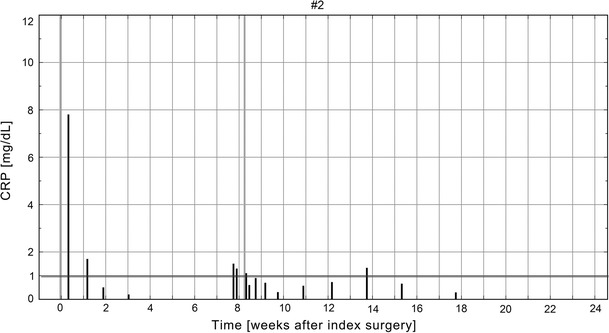
C-reactive protein kinetics of patient #2. Weeks are calculated from the index surgery (THA). The *two grey vertical lines* represent the procedures (THA and ID), while the *grey horizontal line* represents the highest value of the normal CRP range (1 mg/dL)

During the postoperative clinical and laboratory follow-up (Fig. 4), the patient demonstrated an elevated CRP (1.3 mg/dL) 5 weeks after reoperation, associated with suture material extrusion. Frequent wound care allowed complete recovery and renormalization of CRP within 2 weeks, without any further recurrence.

Fourteen months after right hip THA, the patient, satisfied with the previous joint replacement despite the complication, requested left hip THA as originally planned. In order to minimize the risk of foreign-body reaction, a different suture material with no colouring or antibacterial agents was selected (undyed Polysorb™), and the closure was performed using as few and as thin sutures as possible. From the sixth to the ninth postoperative week the patient complained about suture material extrusion through the scar and mild local tenderness, but no blood test abnormalities, ultrasonographically detectable abscess or significant functional impairment occurred. This complication resolved with appropriate wound care. Two years after the first joint replacement and 10 months after the second one, the patient is extremely satisfied with her bilateral THA.

## Discussion

The two cases presented demonstrate that an adverse reaction to an absorbable suture after total hip replacement might determine a clinical condition that cannot be reliably differentiated from a surgical site infection.

Both cases were standard, uncomplicated procedures performed on low-risk patients, had an uneventful early postoperative course with no complaints up to the 8th–9th postoperative week. They then developed local, systemic, US and laboratory signs of surgical site infection. Although no positive cultures were available, ID could not have been questioned or delayed, given the high probability of infection and the negative prognostic impact of the elapsed time [1, 4, 5]. Because of this latter concern, joint aspiration was not attempted and both hips were quickly reoperated. The patients had a mild recurrence 8 and 5 weeks, respectively, after ID, likely because the same suture material was used as in the primary surgery, but in a smaller amount. However, knowledge of the histological diagnosis, awareness of having reused the suture material that elicited the first foreign-body reaction, and the similar presentation and timing suggested that we provide simple wound care, without any surgical or antibiotic treatment, and the relapses self-healed without any consequences.

To the best of our knowledge, only three other cases of adverse reaction to suture material mimicking a periprosthetic joint infection have been reported to date, by Sayegh and coworkers [3] (Table 1). Those three patients received the same resorbable suture as in our series, but without antibacterial agent (Vicryl^®^ instead of Vicryl Plus^®^). Interestingly, the timing is almost identical: 8 and 7 weeks after index sugery in our series (with recurrences 8 and 5 weeks after ID, respectively); 6, 8 and 9 weeks after index surgery in Sayegh et al.’s series (with no mention of possible relapses). On the other hand, the eosinophilia described by Sayegh et al. was not confirmed in our two patients, who showed a normal total WBC count, with minor elevation of neutrophil percentage but normal neutrophil count. Eosinophils were within the normal range for percentage and for count. While the three previously described patients had an extensive involvement of all the layers from the hypodermis to the intracapsular space, our two patients had a relatively superficial involvement, with no penetration of the glutei muscle cuff. We believe that this might depend on capsular repair, which we never performed after a straight lateral approach, but might have been performed by Sayegh and coworkers, especially if a posterolateral approach was used. However, this explanation is conjectural, since surgical approach and capsular repair are not mentioned by the above authors.

**Table 1:** Synoptic table summarizing the main clinical information from the three cases reported by Sayegh et al. [3] (I, II and III) and the two cases presented here (IV and V)

	I	II	III	IV (#1)	V (#2)
Suture material	Vicryl^®^	Vicryl^®^	Vicryl^®^	Vicryl Plus^®^	Vicryl Plus^®^
Presentation time (weeks after surgery)	8	9	6	8	7
Local inflammation	+	+	+	+	+
Draining sinus	+	+	–	+	–
Body temperature (°C)	37.9	37	39	<37	37.5
CRP	Elevated	Elevated	Elevated	Elevated	Elevated
WBC	Normal with eosinophilia	Normal with eosinophilia	Normal with eosinophilia	Normal	Normal
Abscess location	Extensive (from subcutaneous to intracapsular)	Extensive (from subcutaneous to intracapsular)	Extensive (from subcutaneous to intracapsular)	Superficial (prefascial)	Superficial and intermediate (pre- and subfascial, with no extension through the glutei muscles)
Recurrence	Not mentioned	Not mentioned	Not mentioned	Yes (8 weeks later)	Yes (5 weeks later)

The two cases presented are the first suture-related pseudoinfections whose recurrence after absorbable suture material re-implantation is documented. Similar timing but different extents between first episode and recurrence confirm the hypothetical aetiopathogenesis, since the same material was used but in different amounts.

All the reported five patients had the wound closed with coated Vicryl^®^, a synthetic suture material made of Polyglactin 910, which is a copolymer obtained from 90 % glycolide and 10 % l-lactide. Its resorption is completed by hydrolysis within 56–70 days from implantation (which corresponds perfectly to the latency of the psuedoinfection). It is used worldwide in most surgical fields, and recently became available associated with an antibacterial agent, triclosan (Vicryl Plus^®^). Few adverse reactions have been reported to date: Holzheimer described inflammation and occasional sinus discharge in 12 patients after subcutaneous suture with Vicryl^®^ or Vicryl Plus^®^ and skin closure with Dermabond^®^ glue in patients operated for hernia, varicose veins and soft tissue tumors [2]. The complication occurred 3–8 weeks after the index procedure, and only in two patients was an infection demonstrated.

Local inflammation after wound healing is likely under-reported, since suture extrusion is a common and benign complication of surgical wounds, often overlooked by patients and general practitioners. Drake and coworkers [6] clearly demonstrated that this phenomenon depends both on the material (Vicryl is more prone to extrusion than Polysorb) and on the volume (the more knots, the higher the risk). On the other hand, some cases of foreign-body reactions to suture material might have been classified as surgical site infection with false-negative cultures, since histological samples are not routinely collected by all surgeons. It is well known that preoperative culture sensitivity is only fair (0.70 from joint aspiration in infected THA according to Qu et al. [7], and possibly lower from sinus discharge swabs), and even intraoperative culture sensitivity is suboptimal (0.94 according to Spangehl et al. [8]). Dealing with a supposed periprosthetic joint infection with no positive cultures is thus not an exceptional experience for orthopaedic surgeons.

However, in the presented cases several elements make occult infection extremely unlikely: multiple cultures (preoperative swabs, three intraoperative samples and postoperative cultures on drain tips) were negative without any preoperative antibiotic administration, the histological examination found a mixed inflammatory infiltrate with lymphomonocytes prevailing over neutrophils, and the relapses self-healed after complete suture absorption or extrusion.

Remarkably, in our patient #2, who received a subsequent contralateral THA sutured with undyed Polysorb™, made of Lactomer (another glycolide/lactide copolymer) coated with a mixture of a caprolactone/glycolide copolymer and calcium stearoyl lactylate, the absorption phase was not uneventful, although the reaction was milder than after the first surgery. The role of the suture material therefore seems to be important, but likely less important than the patient’s aptitude to foreign-body reaction.

In conclusion, the five cases described to date allow us to define a somewhat novel complication of total hip replacement, the suture-related pseudoinfection (SRPI). SRPI is characterized by local and systemic signs of inflammation occurring 6–9 weeks after THA, when sutures are absorbed. A sterile abscess is usually located in the subcutaneous tissue, with possible superficial seropurulent drainage and deep extension through the fascia. The phenomenon cannot be reliably differentiated from a postoperative infection at the time of its presentation, and only the negative result of all the microbiological samples, the benign course and the histological examination allow the differential diagnosis, which is always ex post. Thus, even though this complication might possibly self-heal after complete absorption of the foreign material, we strongly advice against nonsurgical management, which would surely worsen the prognosis of a true, more common postoperative infection.

The awareness of this exceptional phenomenon leads to some clinical considerations. First, the principle that only early periprosthetic joint infections are eligible for simple irrigation and debridement should not be overemphasized. If strict exclusion criteria were applied [4, 9–12], some of the reported five patients might have been candidates for two-stage revision arthroplasty, since more than 6 weeks had elapsed from implantation and no microbiological diagnosis was available. The acute onset and the short interval from onset to treatment, rather than from implantation to onset, should be considered a relevant positive factor in favour of a prosthesis-sparing surgery. Second, histological specimens should always be collected when potential periprosthetic joint infections are debrided. Third, the smallest possible volume of suture material should be left in every wound, especially in the subcutaneous tissue, where little tensile strength is required and foreign-body reactions seem to be more devastating due to extensive fat necrosis. In our routine surgical practice, deep subcutaneous suture after THA is now obtained with #0 suture only (instead of using two or three #2 stitches), and the number of knots has been reduced from four to three. Fourth, if a SRPI is suspected, closing the wound after ID with a suture material with low propensity to induce foreign-body reaction might lower the chance and the severity of possible recurrences.
